# Advances in Antiviral Therapy for Subacute Sclerosing Panencephalitis

**DOI:** 10.3390/molecules26020427

**Published:** 2021-01-15

**Authors:** Koichi Hashimoto, Mitsuaki Hosoya

**Affiliations:** Department of Pediatrics, School of Medicine, Fukushima Medical University, Fukushima 9601295, Japan

**Keywords:** SSPE, ribavirin, therapy, antiviral agent

## Abstract

Subacute sclerosing panencephalitis (SSPE) is a late-onset, intractable, and fatal viral disease caused by persistent infection of the central nervous system by a mutant strain of the measles virus. Ribavirin intracerebroventricular therapy has already been administered to several SSPE patients in Japan based on fundamental and clinical research findings from our group, with positive therapeutic effects reported in some patients. However, the efficacy of this treatment approach has not been unequivocally established. Hence, development of more effective therapeutic methods using new antiviral agents is urgently needed. This review describes the current status of SSPE treatment and research, highlighting promising approaches to the development of more effective therapeutic methods.

## 1. Introduction

Despite the availability of effective measles vaccination programs, measles remains a major cause of child mortality worldwide. The WHO Global Vaccine Action Plan aims to achieve measles eradication in at least five World Health Organization regions by 2020 [1]. However, the number of measles cases has continued to increase until 2019, with data showing that the number of measles cases in the first three months of 2019 increased by 300% relative to the same period in 2018. Although measles can be almost completely prevented with two safe and effective vaccine doses, global coverage of the first dose of measles vaccine remains stagnant at 85% [2].

Subacute sclerosing panencephalitis (SSPE) is a progressive and fatal neurodegenerative encephalitis caused by persistence of the measles virus in the central nervous system (CNS). Since the growth of SSPE virus is thought to be directly involved in neuropathy in SSPE, treatment with antiviral drugs and immunostimulants that suppress virus growth has been attempted. This review discusses various prevalent approaches and new avenues in SSPE antiviral therapy.

## 2. Subacute Sclerosing Panencephalitis (SSPE)

### 2.1. Clinical Features and Epidemiology of SSPE

SSPE is a slow viral infection caused by a mutated measles virus (SSPE virus). This disease develops approximately 2 to 10 years after the onset of measles, causing diminished intelligence, personality changes, bradykinesia, etc. Subsequently, cerebral function is progressively impaired, leading to severe dementia, vegetative state, and eventually, death [3]. The diagnosis of SSPE is made primarily via analyzing clinical symptoms, neurological findings, and laboratory findings such as blood, cerebrospinal fluid, electroencephalograms, and imaging tests [4]. SSPE often progresses subacutely, and the Jabbour classification, which describes the characteristic medical conditions seen at different stages, is used to divide the progression of the disease into four stages [5] and classify patients accordingly. The neurological disability index [6] is used to determine the therapeutic effects of various treatments on SSPE. The progression of SSPE may take different courses, some of which involve a fulminant form of rapid progression accompanied by a severe prognosis [7]. On the contrary, there are also cases with good prognosis, such as patients who exhibit slow progression over 10 years or more, patients who show repeated chronic recurrence and remission, and a small number of patients that display spontaneous improvement in symptoms and are able to walk again after being bedridden [8]. Risk et al. reported the natural history of 118 patients with SSPE in the Middle East [9]. Among these SSPE patients, 40% died within 1 year of onset, 19% died within 2 years, and 41% survived for more than 2 years. An additional 5% died within 3 months, while 20% survived for more than 4 years. Fifty-three percent of patients experienced noticeable improvements, remissions, or plateaus. However, SSPE is fatal in most cases.

Since the prognosis of SSPE is poor, the best means of controlling this disease is by preventing its occurrence. The number of SSPE patients is positively correlated with the number of measles patients [10], and the incidence of SSPE is inversely proportional to the rate of measles vaccination. This has been demonstrated in high-income countries where the prevalence of SSPE has been steadily declining since the introduction of the measles vaccine in the 1960s [11]. While the exact incidence of SSPE among patients with measles is unknown, Bellini et al. concluded that 6.5–11 individuals in every 100,000 cases of measles are at risk of SSPE [12]. However, a greater risk of onset of SSPE is seen in individuals infected with spontaneous measles at a younger age. For instance, a German study found that 1 in 1700 to 1 in 3300 measles patients under 5 years of age were at risk of developing SSPE. Furthermore, the risk of developing SSPE was 1.7 times higher in measles patients under the age of 3 than in those under the age of 5 [13]. Similarly, a study in Japan showed that the risk of developing SSPE was 11.2 times higher for measles patients under 2 years of age than for those over 2 years of age. In addition, the risk of developing SSPE was three times higher in infants with measles within 12 months of birth compared to those within 12–24 months [10]. Another study in California found that the incidence of SSPE after measles was 1 in 1367 in children under 5 years and 1 in 607 in children under 1 year [14]. Thus, the risk of incidence of SSPE in measles patients is clearly higher in younger children, and especially high in infants.

Moreover, individuals with SSPE are more likely to have had measles during infancy. In a study of 350 SSPE patients in the United States, 292 patients (83.4%) had a history of spontaneous infection of measles and were more likely to have had measles under the age of 2 (24% under 12 months and 22% at 12–24 months). Of the 58 patients with no history of measles, 40 had been vaccinated with the live measles vaccine [4]. In the early days of the measles vaccine in the United States, there were rare reports of SSPE. However, it was reported that most of these patients had subclinical spontaneous measles before vaccination and were not affected by the vaccine strain [15]. Bellini et al. analyzed the vaccine association in 11 patients who developed SSPE between 1992 and 2003, 9 of whom had been vaccinated against measles. Wild-type measles was detected in all 11 cases, confirming that the genotype responsible for infection was different from that of the vaccine strain [12].

### 2.2. Etiology and Virological Characteristics of SSPE

In 1933, a report by Dawson suggested that SSPE is caused by a virus [16]. Subsequently, in the 1960s, the measles virus was isolated from a mixed culture of brain cells of SSPE patients and measles virus-sensitive cells, clearly showing the association between SSPE and the measles virus [17,18]. However, the virus could not be isolated from cell-free clinical specimens, suggesting that SSPE is caused by persistent infection of the measles virus in the brain and that the measles virus in SSPE spreads between nerve cells without releasing virus particles [19]. Moreover, some reports suggested that the virus spreads between nerve cells through a microfusion at the synaptic membrane [20,21].

**Figure 1:**
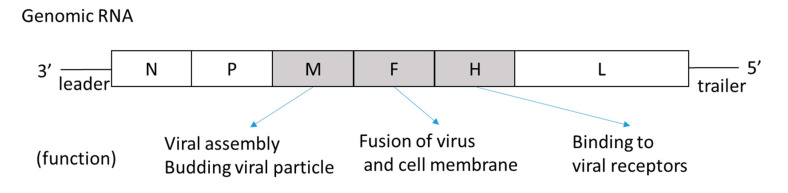
Measles virus genome showing the most common mutations found in SSPE cases. Genes that are mutated in many SSPE viruses compared to wild measles viruses are shown in gray.

**Figure 2:**
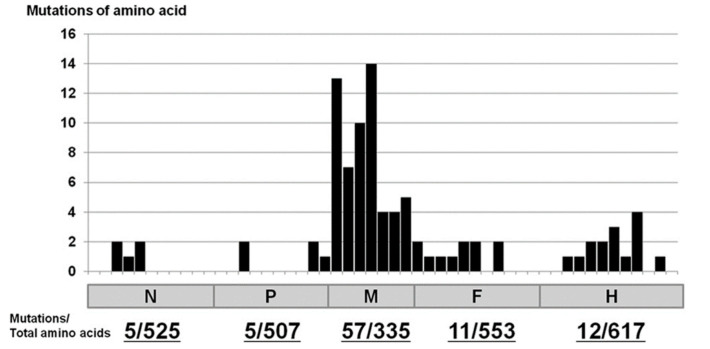
Amino acid mutations in viruses derived from brains of mice with persistent. Measles Virus infection. The genes are listed on the horizontal axis. Each horizontal axis tick represents 50 amino acids. The number of amino acid mutations is presented on the vertical axis. Among the 10 clones, when any 1 clone had an amino acid mutation at a position, it was counted as one mutation. Overlapping mutations at the same position in multiple clones were counted as a single mutation [33].

The measles virus belongs to the Morbillivirus genus within the family *Paramyxoviridae* and the order *Mononegavirales*. This enveloped virus produces pleomorphic virus particles with size up to 900 nm, and an average size of 150–300 nm [22]. Its genome is a single-stranded RNA of 15,894 nucleotides, encoding 6 structural proteins—nucleocapsid (N) protein, phosphorylated (P) protein, matrix (M) protein, fusion (F) protein, hemagglutinin (H) protein, and polymerase (large, L) protein. Two non-structural proteins, V and C, are produced from the P gene [23], and these interact with the host immune system to alter its sensitivity and responsivity [24,25,26,27]. While the M protein plays a role in viral assembly and the budding of infectious viral particles, the H and F proteins form the viral fusion complex involved in the entry of the virus into the host cell. The H protein helps the virus bind to the entry receptor, and the F protein mediates the fusion of the virus and the host cell membrane (Figure 1). Furin protease cleaves the F0 precursor into F1 and F2 subunits, which undergo changes in their three-dimensional structure to form a trimer. The extracellular domain is composed of F1 and F2 subunits containing a fusion peptide at the *N*-terminus, followed by seven repeating domains complementary to the *N*-terminus and *C*-terminus, respectively [28,29]. The measles virus isolated from the brains of SSPE patients exhibits mutations specific to the M, F, and H genes (especially the M gene) (Figure 1) and is characterized by neurocompatibility, neuropathogenicity, and a lack of viral particle-forming ability [21,30,31,32]. In-vivo studies using nude mice inoculated in the brain with the measles virus have also reported the accumulation of gene mutations, especially in the M gene, during persistent infection (Figure 2) [33]. On the other hand, recent studies have shown that many virus strains isolated from SSPE patients have substitutions in the extracellular domain of the F protein, resulting in improved fusion activity. Measles virus with hyperfusogenic mutant fusion proteins spreads between human neurons in a cell-to-cell manner. Both H protein and hyperfusogenic F proteins have been shown to play important roles in measles virus diffusion between human neurons [21]. Moreover, the nucleotide sequence of the SSPE virus isolated from the brains of SSPE patients corresponded to the nucleotide sequence of the wild measles virus strain that patients were originally infected with when they suffered from measles, except for some specific genetic mutations [34,35]. Wild-type measles virus strains infect target cells using signaling lymphocyte activation molecule 1 (also called SLAMF1, SLAM or CD150) and Nectin-4 receptor [36,37,38]. The vaccine strain of measles virus infects cells in vitro via the CD46 molecule [39,40]. However, these molecules are not expressed in the central nervous system; therefore, the mechanism by which the measles virus infects nerve cells is unclear.

On the contrary, some reports suggest the involvement of host factors in the onset of SSPE. For instance, children infected with measles before the age of two are at an increased risk of developing SSPE [10]. It is speculated that persistent infection in the brain is more likely to occur if measles virus infects children before the immune system and central nervous system are fully developed. Moreover, some studies have reported associations between SSPE and single nucleotide polymorphisms (SNPs) of genes in the host immune system. These include SNPs in genes related to innate immunity such as MxA [41], toll-like receptor 3 (TLR3) [42], and TLR4 [43], and in those related to acquired immunity such as interleukin-2 (IL2) [44], IL4 [45], IL17 [43], IL18 [46], granzyme B (GZMB) [47], and programmed cell death 1 (PDCD1) [48]. Quantitative or qualitative differences in immune response to the measles virus caused by these SNPs may be related to disease susceptibility to SSPE. Thus, the development of immune or central nervous system and genetic variation in immune responses may be involved in the onset of SSPE.

## 3. Treatment

Since the incidence of SSPE is extremely low in developed countries, randomized controlled trials on SSPE with large sample sizes have not been reported. Nevertheless, therapies involving oral administration of inosine pranobex and intraventricular administration of interferons have been attempted and proven to be effective in a relatively large number of cases.

### 3.1. Inosine Pranobex

Inosine pranobex (IP), a combination of inosine, acetamidobenzoic acid and dimethylamino isopropanol, is a drug that exerts both antiviral and immunostimulatory effects [49]. The exact mechanism of the antiviral effect has not yet clearly defined, but it is hypothesized that inhibition of viral RNA synthesis is due to modification of the structure of infected cell ribosomes by the drug component of IP and rapid metabolism of IP. IP has an antiviral effect in vitro on several RNA and DNA viruses, but has not been observed on measles virus [50]. As immunomodulatory effects, T cell proliferation, NK cell activation, and increased production of inflammatory cytokines (e.g., IL-2, IFN-γ) have been observed in vitro and in vivo. Due to its antiviral effect, immunomodulatory effect, and safety, it has been used in SSPE, herpes simplex virus, human papillomavirus, human immunodeficiency virus, influenza virus, and airway virus infections, cytomegalovirus, and Epstein-Barr virus infections [49]. Generally, 50–100 mg/kg per day is orally administered to SSPE patients in three or four divided doses. Clinical symptoms of SSPE improved or stopped progression in 33% (5/15) [51], 11% (2/18) [52], and 66% (10/15) [6] of patients subjected to IP treatment in different studies. However, since the spontaneous remission rate without IP was reported to be 4–10%, the effect of IP on SSPE is unclear. IP also prolonged the survival of SSPE patients (*p* < 0.01), as the 8-year survival rate of 98 patients who received IP was 61% compared to the survival rate of 8% seen in patients who did not receive this treatment [53].

### 3.2. Interferons

Interferons are naturally produced by animal cells as a defense mechanism, and are used in the treatment of SSPE due to their antiviral activity. One to three million units of interferon (IFN) (α or β) is typically administered intrathecally or intraventricularly one to three times a week to treat SSPE. IFNs have also been reported to be effective when used in combination with IP. Yalaz et al. reported improvement of symptoms in 50% of patients (11/22) when intraventricular IFNs were delivered with IP [54], while Gascon et al. reported improvement in 17% (3/18) and stabilization of symptoms in 28% (5/18) of patients orally administered IP monotherapy [55]. As is the case with IP monotherapy, the efficacy of IFN monotherapy is uncertain though administration of IFN has been reported to stop progression more often than in patients not subjected to treatment. However, follow-up of the cases reported by Yalaz et al. [54] for an additional 5–9 years revealed that 8 of the 11 cases that had improved and all 5 cases that had stopped progression subsequently showed neurological regression, and 7 of the 13 patients with worsening symptoms died. Therefore, the therapeutic effect appears to be temporary and does not result in improvement of long-term prognosis [56]. In a report comparing IP alone with IP in combination with IFN intracerebroventricular therapy, there was no significant difference in the rate of improvement in or arrest of progression of symptoms (34% and 35%) between the two groups. However, these rates are higher than that in patients who received no treatment [57]. Although IFN α is useful for treatment, side effects include fever, lethargy, loss of appetite, and chemical meningitis [58]. In addition, the effect on central nervous system function as interferonopathy has also been reported [59]. Most patients treated with intraventricular IFN and oral IP do not show serious side effects, but long-term repeated treatments carries the risk of developing meningitis, IFN α-induced encephalopathy, and upper and lower motor neuron toxicity [60].

### 3.3. Ribavirin and Research on Treatment Methods

In search for alternatives, we screened for drugs showing anti-SSPE effects in vitro and identified the nucleic acid analogs pyrazofurin, 6-azauridine, 3-deazaguanine, and ribavirin as candidate drugs [61]. Ribavirin exhibits weaker anti-SSPE activity in vitro than pyrazofurin, 6-azauridine, and 3-deazaguanine as an antiviral drug, but it has been shown to be effective for treatment of respiratory syncytial virus [62,63], influenza virus [64,65], and Lassa fever virus [66,67] infections. Intravenous administration of ribavirin has also been reported to be effective against measles pneumonia [68,69]. However, no effect was observed in SSPE patients upon oral administration of ribavirin, probably because concentrations of this drug in the cerebrospinal fluid concentration did not reach the levels that exhibited the anti-SSPE effect in vitro [70].

However, antiviral effects of ribavirin against SSPE have been observed in animal experiments. Daily intracerebral administration of ribavirin to hamsters that ingested a lethal dose of SSPE virus improved survival rate in a dose-dependent manner [71] and increased ribavirin concentration in the brain in a dose-dependent manner, such that it reached the concentrations that were effective against the SSPE virus in vitro [72]. Based on in-vitro and in-vivo studies using hamsters, the minimum inhibitory concentration of ribavirin against SSPE virus in the brain was estimated to be 5–10 µg/mL (20.5–40.9 µM) and the complete inhibitory concentration was estimated to be 50–100 µg/mL (204.8–409.5 µM) [61,72]. However, ribavirin concentration in the cerebrospinal fluid (CSF) needs to be monitored because the toxic concentration of ribavirin is close to its effective concentration [61]. Therefore, the target concentration of ribavirin in CSF of SSPE patients was set to 50–200 µg/mL Furthermore, the combined effect of ribavirin and IFN-α has been demonstrated both in vitro and in vivo using hamsters [73].

Hence, high-dose intravenous administration of ribavirin was attempted in human SSPE patients as well, based on the report that blood ribavirin is transferred to the central nervous system via the blood-brain barrier [74]. The minimum effective concentration of ribavirin in the cerebrospinal fluid was reached by intracerebroventricular administration of IFN and intravenous administration of the maximum tolerated dose of ribavirin [75,76]. Although the number of test cases was small, clinical symptoms (neurological disability index score) improved and measles antibody titer in cerebrospinal fluid decreased in these patients [76,77]. However, systemic administration of high doses of ribavirin caused hemolytic anemia, and SSPE relapsed when treatment was discontinued.

Therefore, administration of ribavirin directly into the ventricle, similar to IFN, was attempted to maintain concentrations of ribavirin in the brain sufficient for eliciting its inhibitory effects on the SSPE virus. Ribavirin was administered intraventricularly using an Ommaya reservoir. The concentration of ribavirin in CSF was maintained at a concentration that completely suppresses the growth of SSPE virus by administering 1–3 mg/kg per day of ribavirin directly into the ventricles one to three times a day [78,79]. While Tomada et al. used daily intraventricular administration of ribavirin for more than 2 months [78], Hosoya et al. used a similar protocol, but with 10 days of daily administration of ribavirin followed by 20 days of drug holidays, or 5 days of daily administration of ribavirin followed by 10 days of drug holidays as one course of treatment [79]. Tomada et al. conducted these trials on 10 patients at various stages of SSPE; seven of them showed improvement in clinical symptoms or decreased measles antibody titer in CSF [78]. Among the five patients that were subjected to ribavirin treatment in Hosoya et al., four showed improvement in symptoms [79]. In both studies, patients who started treatment in the earlier stages of SSPE showed clearer improvement in symptoms and decreased measles antibody titers in CSF [78,79]. However, intraventricular administration of ribavirin resulted in moderate or transient side effects such as lip/gingival swelling, conjunctival hyperemia, headache, and a tendency for somnolence. Although anemia is a common side effect of systemic administration of ribavirin, this was not seen with intracerebroventricular administration.

**Figure 3:**
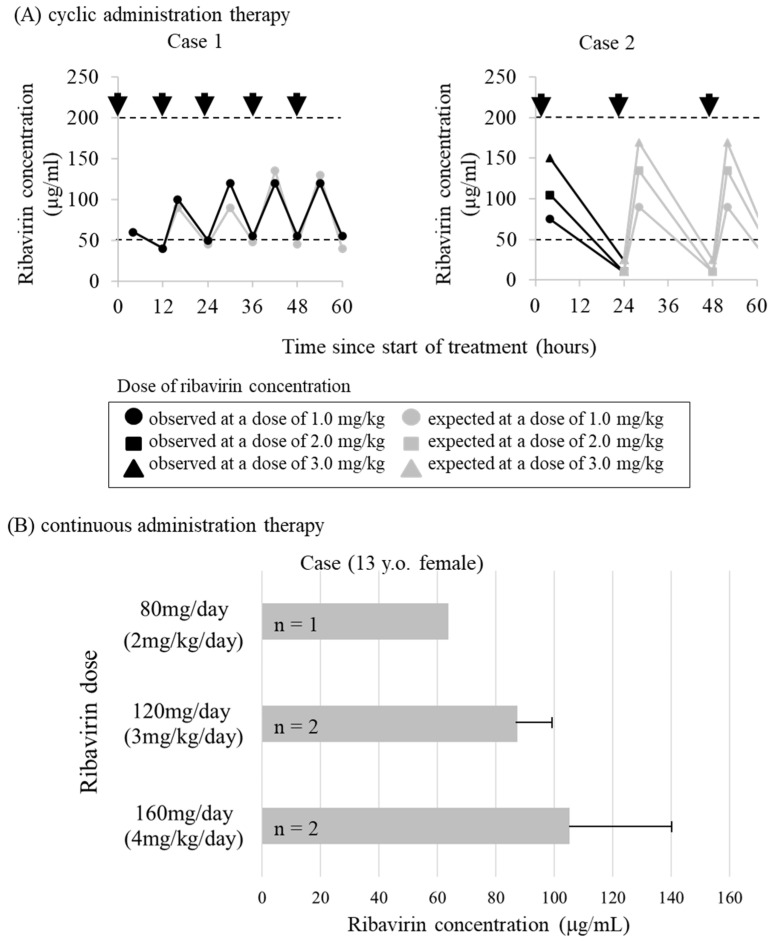
Ribavirin concentration in the cerebrospinal fluid. (**A**) Expected and observed ribavirin concentrations in the cerebrospinal fluid (CSF) following repeated intraventricular administration (cyclic administration therapy). In case 1, ribavirin concentrations in the CSF were maintained throughout at levels sufficient for complete inhibition, but this was not done in case 2. The arrows indicate points of time when ribavirin was administered. CSF was collected by lumbar tap just before ribavirin administration [79]. (**B**) Ribavirin concentrations in the CSF following continuous intraventricular administration (continuous administration therapy). CSF was collected by lumbar tap 3–10 days after the start of ribavirin administration or at the time of saline substitution. Bars show the mean ribavirin concentration and standard error. Representative case is shown [82].

However, it was difficult to achieve the effective concentration of ribavirin in CSF in some cases by the intraventricular administration method through the Ommaya reservoir [79] (Figure 3A). Such differences in ribavirin concentrations in CSF across individuals may depend on individual differences in CSF clearance and volume [79,80]. Moreover, complications related to bacterial meningitis have been reported due to frequent puncture of the Ommaya reservoir [81].

**Figure 4:**
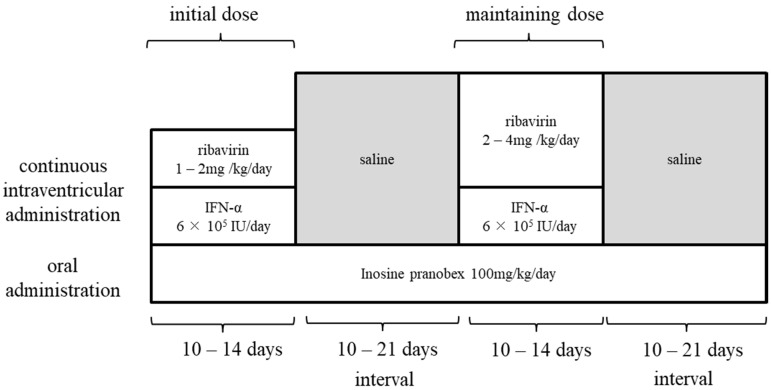
Protocol for ribavirin continuous infusion therapy using a continuous infusion pump. Patients were given ribavirin and interferon-α using a subcutaneous continuous infusion pump with oral administration of inosine pranobex (continuous administration therapy). At the start of the treatment, 1–2 mg/kg per day was used as the initial ribavirin dosage. Ribavirin dosage was raised according to its concentration in CSF and treatment cycles were repeated as maintenance doses [82].

To maintain effective concentrations in CSF and to avoid frequent puncture during bolus administration (single repeated administration), continuous administration of ribavirin into the ventricles (continuous infusion therapy) by a subcutaneous implantable continuous infusion pump (Archimedes^®^, Codman, Germany) was attempted. This pump had been previously used for intrathecal administration of baclofen as treatment for spastic paraplegia [83]. The treatment protocol with ribavirin involved continuing oral administration of inosine pranobex, with ribavirin and INF for 10–14 days, and drug holidays for 10–21 days (Figure 4). The continuous intracerebroventricular infusion therapy was attempted in three SSPE patients, one of which was discontinued at the request of the family due to exacerbation of symptoms. In all patients, the target concentration of ribavirin in CSF reached 50 to 200 µg/mL at doses of 1 to 3 mg/kg per day (Figure 3B). Although the disease had progressed to advanced stages in all three patients, the patients survived for more than 5 years since the start of continuous infusion therapy, the disease remained in stage III, and the progression was slower except in the discontinued case [82]. Although SSPE causes many patients to die within a few years of onset, this long-term survival is thought to be the result of improved medical support as well as the therapeutic effects of continuous infusion therapy. In addition, continuous infusion therapy can be performed once or twice a month, and patients can spend their time at home even during treatment, leading to improved quality of life of patients and their families. The intracerebroventricular ribavirin administration therapy for SSPE was carried out with the approval of the ethics review board of the university and with the consent of patients or their substitutes.

### 3.4. Other Existing Clinical Drugs

Although existing clinical drugs such as amantadine, steroids, cimetidine, and antiepileptic drugs have shown therapeutic effects against SSPE, their efficacy is case-dependent and has not been clearly established [84,85]. Moreover, viral polymerase inhibitors such as remdesivir [86] and favipiravir [87,88] could be candidates for treatment of SSPE. Remdesivir specifically inhibits polymerases of various RNA viruses, such as phyllovirus, henipavirus, and coronavirus, and exhibits broad spectrum activity against paramyxovirus infections, including those caused by measles virus [86]. Pharmacokinetic studies conducted in non-human primates have shown that remdesivir and its derivative active nucleosides can reach the brain [89]. In addition, favipiravir, which was developed as an anti-influenza drug, showed antiviral activity against a wide range of RNA viruses [87,88] and showed inhibitory effects on the in vitro growth of the SSPE virus; these effects were similar to those of ribavirin [90]. Furthermore, recent in vitro and in vivo studies on severe febrile thrombocytopenia syndrome showed that favipiravir had higher antiviral activity than ribavirin [91]. Since it is not clear which clinical drugs can be used to treat SSPE, we recommend that the effects of these drugs must be confirmed through further studies. Moreover, when using intravenous and oral administration, it is important to develop injectable drugs with sufficient dosage so that they can enter the cerebrospinal fluid.

### 3.5. Preclinical Drugs

Some RNA synthesis inhibitors and entry/fusion inhibitors have been reported as compounds with anti-measles virus or anti-SSPE virus activity. Compound 16677 (1-methyl-3-trifluoromethyl-5-pyrazolecarboxylic acid), which is a viral RNA synthesis inhibitor, and its analogs AS-136A and 2O (ERDRP-00519) show high anti-measles virus activity in vitro [92,93,94]. Invasion/fusion inhibitors exhibit antiviral activity by inhibiting continuous structural changes in F proteins or interaction with receptors to prevent viral entry. The receptor binding site for measles virus H is considered a potential neutralization target. Neutralizing antibody-derived molecules such as single-strand variable fragments that target the H protein are possible candidates for treatment of SSPE [95]. In addition, small molecules such as the fusion-inhibiting peptide Z-d-l-Phe Gly [96] and AS-48 and 3G (analog of AS-48) inhibit membrane fusion in vitro [97,98]. Furthermore, seven C-terminal repeating domains (HRC) of the F protein and complementary peptides of HRC suppressed viral growth in the brain and prevented mortality in animal studies [99,100]. Thus, these molecules may also be considered for their therapeutic potential in SSPE.

## 4. Conclusions

Since the incidence of SSPE is low in developed countries, evaluating the efficacy of drugs against SSPE using comparative studies with a control drug is difficult. In addition, the clinical course of SSPE varies from case to case, and therefore, the effectiveness of antiviral therapy and changes in prognosis must be carefully determined by long-term observation in a larger number of cases. Our studies suggest that intraventricular administration of ribavirin resulted in a clear improvement in symptoms in patients who started treatment in the early stages, indicating the importance of early diagnosis and treatment of SSPE. Clearly, drug development for SSPE is an urgent issue since none of the currently available drugs can cure SSPE. Hence, improving the measles vaccination rate and eradicating measles are the best strategies for controlling and preventing the onset of SSPE. However, new treatment approaches using molecules that display potential against SSPE must also be explored, with special focus on developing oral or intravenous drugs that can penetrate the blood-brain barrier and show desired anti-SSPE activity.

## Data Availability

Not applicable.
